# AI in Psychiatry for Improving Continuity of Patient Care: Protocol for a Mixed Methods Systematic Review

**DOI:** 10.2196/95931

**Published:** 2026-08-05

**Authors:** En Jie Tan, Wen Jie Dominic Yao, Xin Er Ong, Jireh Foo, Andrew Ian-Hong Phua, Maria Abraham

**Affiliations:** 1 Institute of Mental Health Singapore Singapore; 2 Saw Swee Hock School of Public Health National University of Singapore Singapore Singapore; 3 National Preventive Medicine Residency Programme National University Health System Singapore Singapore; 4 Lee Kong Chian School of Medicine Nanyang Technological University Singapore Singapore; 5 Population Health and Integrated Care Sengkang General Hospital Singapore Singapore

**Keywords:** artificial intelligence, psychiatry, continuity of patient care, community mental health services, machine learning

## Abstract

**Background:**

Continuity of care is essential in psychiatric services due to the chronic, relapsing nature of mental health conditions, yet care pathways remain heavily fragmented at critical transition points. Although advancements in AI and machine learning (ML) offer powerful capabilities to track longitudinal data and automate clinical decision-making, a structured appraisal of their efficacy in supporting continuity of psychiatric care is lacking. This protocol outlines a mixed methods systematic review to evaluate how AI-driven workflows can proactively enhance monitoring, optimize triage and care resource allocation, and address systemic coordination gaps.

**Objective:**

The primary objective of this systematic review is to evaluate the effectiveness of AI and ML interventions in psychiatric care settings in improving the continuity of patient care. Secondary objectives include stratifying the types of AI architectures used and identifying implementation barriers and facilitators.

**Methods:**

A systematic literature search of MEDLINE, Embase, CENTRAL, CINAHL, and APA PsycInfo will be conducted to identify peer-reviewed randomized controlled trials, nonrandomized interventional studies, and qualitative or mixed methods evaluations published between January 1, 2016, and December 31, 2025. Two independent reviewers will perform study screening, data extraction, and quality assessment. A mixed methods convergent synthesis using the Joanna Briggs Institute (JBI) convergent segregated approach will be carried out to synthesize quantitative evidence on effectiveness and qualitative data on implementation.

**Results:**

This review is self-funded and was officially registered with PROSPERO on January 24, 2026 (CRD420251245352). Comprehensive database searches have commenced, with full-text screening and transcript reviews projected to conclude by late August 2026, followed by data analysis and submission of the systematic review manuscript targeted for early spring 2027.

**Conclusions:**

By systematically mapping interventions across patient, institutional, and health system levels, this review will clarify the clinical effectiveness, ethical boundaries, and logistical implementation factors of psychiatric AI tools. Ultimately, these consolidated insights will provide an evidence-based foundation to inform clinical guidelines; governance frameworks; and the design of proactive, learning mental health systems.

**Trial Registration:**

PROSPERO CRD420251245352; https://www.crd.york.ac.uk/PROSPERO/view/CRD420251245352

**International Registered Report Identifier (IRRID):**

PRR1-10.2196/95931

## Introduction

### Background

#### Continuity of Care: Importance and Challenges

Continuity of care refers to the degree to which a series of health care services are experienced by patients as coherent, connected, and consistent with their medical needs and personal circumstances [1-3]. It encompasses informational continuity (shared data across health care providers), relational continuity (stable patient-provider relationships), and management continuity (coordination across care settings) [4-6]. Effective continuity of care has been linked to improved patient satisfaction, better health outcomes, and reduced health care use, particularly for individuals requiring long-term management or multidisciplinary support [7-11].

Despite its importance, ensuring continuity of care remains a persistent challenge. Fragmentation in health systems, barriers to data sharing, and transitions between health care providers often disrupt seamless patient management [12-16]. Discontinuities are particularly frequent at the interface between primary, secondary, and community care. Limited interoperability of electronic health records, variations in clinical practices, and resource constraints further impede coordinated care delivery [15-18]. Consequently, patients may experience inconsistencies in information exchange [19], duplication of tests [20,21], or complete disengagement from necessary follow-up care [14,22-24].

#### Layered Complexities in the Field of Psychiatry

Continuity of care poses additional complexities in psychiatry, where patients often belong to vulnerable populations requiring sustained, holistic management [25-27]. Mental health conditions are characterized by chronicity; fluctuating symptoms; and the interplay of biological, psychological, and social factors [28-32]. Disruptions in care continuity—for instance, due to health care provider turnover, care transitions, or inadequate follow-up—can lead to relapse, hospitalization, or disengagement from treatment [33-39]. The stigma surrounding mental illness, along with insufficient integration between psychiatric and general health services, further compounds these challenges [40,41]. As psychiatric care heavily depends on therapeutic relationships and consistent communication, fragmented care delivery has particularly adverse implications for patient well-being [42-47].

#### Advancements in AI and Automation of Work Processes

Over the past decade, AI has evolved from a niche technological pursuit into a transformative force with the unprecedented capacity to handle complex data, recognize patterns, and automate decision-making processes. In health care, this automation extends beyond administrative functions to diagnostic support, personalized treatment recommendations, and longitudinal patient monitoring [48-51]. AI applications have increasingly focused on patient management through tools that enable data-driven clinical decision-making, early detection of health risks, and adaptive care coordination [52]. Predictive algorithms analyze large-scale patient data to identify potential deterioration in health status, while conversational AI tools facilitate continuous patient engagement beyond clinical settings. AI-powered systems also streamline information exchange among multidisciplinary teams, contributing to more coherent and responsive care pathways. These technologies pave the way for a model of proactive and personalized health care, in which patient interactions are not episodic but are sustained through data connectivity and intelligent automation. The integration of AI-driven automation has thus become central to improving efficiency, reducing clinician workload, and enhancing the precision of care delivery [52-59].

#### The Potential of AI-Augmented Workflows for Continuity of Care in Psychiatry

Within psychiatry, AI offers promising avenues to strengthen continuity of care by enhancing information flow, patient monitoring, and clinical decision-making. Machine learning (ML) algorithms can track longitudinal patterns in patient data to flag early signs of relapse or risk behaviors, allowing timely interventions. AI-driven chatbots and mobile mental health apps support patient engagement, bridging gaps between appointments and promoting adherence to treatment plans [45,47,60,61]. Furthermore, AI can facilitate integration across care settings by synthesizing multimodal data from electronic health records, therapy notes, and wearable devices (digital phenotyping) and recognizing subtle patterns and longitudinal trends that may be missed even by experienced health care professionals, thereby generating cohesive, patient-centered insights. By automating routine tasks and augmenting human oversight, AI removes the necessity for clinicians to provide constant manual oversight for every individual in their caseload, thereby supporting systems facing acute staff shortages. This allows the system to “triage” human attention to patients flagged by AI as being at high risk or in crisis, thereby fostering more continuous, personalized, and coordinated psychiatric care [62-64]. Nevertheless, recent work has similarly emphasized that AI systems used for conversational or risk-oriented support in health care settings should be evaluated not only for task performance but also for safety-sensitive reasoning and oversight requirements [65].

Despite a growing number of studies in this area, a rigorous synthesis of the evidence is needed. This systematic review aims to comprehensively evaluate the effectiveness and implementation factors of AI and ML interventions specifically designed to improve the continuity of patient care in psychiatric care settings, thereby contextualizing the current state of knowledge for researchers, clinicians, and policymakers [45,66,67].

### Objectives

The primary aim of this study is to evaluate the effectiveness of AI and ML applications in enhancing the continuity of psychiatric care compared to conventional standard-of-care protocols. The secondary objectives are to systematically categorize the diverse typologies and functional architectures of AI-driven tools deployed in mental health services and synthesize patient-reported outcomes (PROs) to gain a nuanced understanding of the patient experience of AI-mediated care. Although quantifying the clinical effectiveness of these AI-ML interventions is paramount, psychiatric service delivery is deeply dependent on workflow integration and stakeholder trust. Therefore, this mixed methods systematic review aims to evaluate both the quantitative efficacy of these tools and the qualitative evidence regarding their implementation barriers, acceptability, and ethical challenges [68].

## Methods

### Guidelines and Registration

This review protocol is informed by the Cochrane Handbook for Systematic Reviews of Interventions and the PRISMA-P (Preferred Reporting Items for Systematic Review and Meta-Analysis Protocols) 2015 [69,70]. Our review is registered with the International Prospective Register of Systematic Reviews (PROSPERO CRD420251245352). Any modifications to our protocol will be formally recorded as amendments in PROSPERO.

### Eligibility Criteria

The inclusion and exclusion criteria are defined using the population, intervention, comparator, outcome, and study design (PICOS) framework [71,72], encompassing both quantitative and qualitative study designs (Textbox 1).

**Inclusion and exclusion criteria for study selection based on the population, intervention, comparator, outcome, and study design framework.**

**Population**

The study population is restricted to adult patients (aged ≥21 years) with a primary psychiatric diagnosis. Pediatric and adolescent populations will be excluded from this analysis due to the distinct models of care inherent to pediatric psychiatry, where continuity of care is significantly mediated by parental or guardian influence. Focusing on the adult population will allow this review to mitigate the confounding variables associated with caregiver-driven health care navigation, thereby ensuring a more homogeneous assessment of AI-integrated care models.

**Intervention**

Eligible interventions must directly leverage AI or machine learning (ML) architectures to sustain or augment psychiatric care continuity, defined as efforts to build coherent, connected care consistent with patients’ medical needs and personal circumstances. Interventions will be classified a priori into 4 mutually exclusive domains, encompassing predictive risk stratification models engineered to proactively predict clinical deterioration, relapse, or psychiatric readmission risk; conversational agents and chatbots using natural language processing or large language model architectures to assist with postdischarge symptom monitoring, treatment compliance tracking, or therapeutic behavioral engagement; continuous passive digital monitoring tools designed to track behavioral phenotypes, sleep disruptions, or kinetic signals via consumer wearables or smartphones; and cross-interface clinical decision support systems that facilitate cross-setting clinical oversight and care coordination recommendations for multidisciplinary teams during care handovers. Conversely, purely administrative automated tools, such as automated SMS appointment reminders or simple scheduling algorithms lacking ML parameters, will be excluded. Furthermore, conventional predictive or risk stratification tools using deterministic scoring matrices, static clinical rating scales, or traditional regression-based risk scores lacking active ML parameters will also be excluded.

**Comparator**

AI interventions will be compared against standard psychiatric care and existing continuity-of-care frameworks. These comparators include established service delivery models without AI augmentation, such as intensive case management, assertive community treatment, and integrated care coordination. Additionally, traditional administrative protocols—specifically manual telephonic follow-ups, coordinated discharge planning, and non-AI digital automation (eg, basic electronic alerts or standardized SMS reminders)—will serve as the primary benchmarks.

**Outcome**

The primary outcomes pertaining to continuity of patient care will be categorized across 3 domains: service continuity outcomes, which evaluate care transition interface fidelity, including 30-day and 90-day psychiatric rehospitalization or readmission rates, emergency department presentations, data transmission latency, and outpatient care scheduling gap durations; clinical outcomes, encompassing standardized adjustments in clinical psychiatric scores alongside documented relapses or clinical decompensation episodes; and patient-reported outcomes, comprising evaluative measures assessing therapeutic alliance matrices between patients and health care providers, treatment satisfaction scores, digital equity parameters, and user-experienced care coordination indices. Secondary outcomes will encompass implementation barriers and facilitators for AI interventions deployed in these domains.

**Study design**

Quantitative study designs will include randomized controlled trials, quasi-randomized trials, nonrandomized controlled trials, and prospective and retrospective cohort studies that use a defined comparison or control group. To capture the lived experience and systemic nuances of AI implementation, qualitative research—specifically studies using thematic analysis or phenomenological approaches—will also be included. Mixed methods studies will also be included to triangulate quantitative efficacy with qualitative insights. The scope will be limited to peer-reviewed journal articles published in the English language to ensure the rigor and accessibility of the analyzed data. Studies will also be restricted to those published in English due to translation capacity constraints.

### Information Sources

To identify all potentially eligible studies, a comprehensive search was conducted across a broad range of biomedical, psychological, and multidisciplinary databases to ensure a thorough synthesis of the literature at the intersection of health care and technology. The following electronic databases were searched for records published between January 1, 2016, and December 31, 2025: Embase (via Elsevier), MEDLINE (via PubMed), the Cochrane Library (including the Cochrane Central Register of Controlled Trials [CENTRAL]), CINAHL (via EBSCOhost), and APA PsycInfo (via Ovid).

The search period was restricted to the post-2015 era to account for the rapid pace of innovation in ML, natural language processing, and computational capabilities. AI applications and technologies developed more than a decade ago were considered likely to be obsolete and unlikely to accurately reflect the current state of the field [52-54].

In accordance with Cochrane recommendations for identifying unpublished research, supplemental searching was performed. This included a manual screening of the reference lists of all included articles and relevant systematic reviews to identify additional primary studies. Furthermore, clinical trial registries, specifically ClinicalTrials.gov and the World Health Organization International Clinical Trials Registry Platform (ICTRP), were searched to ensure the inclusion of recent research efforts.

### Search Strategy

The search strategy was designed to maximize sensitivity and was developed in collaboration with a medical information specialist (Textbox 2). A pilot search strategy was first established for PubMed and subsequently translated for each database using database-specific controlled vocabularies, including MeSH for the Cochrane Library, Emtree for Embase, and CINAHL Headings for CINAHL. Keywords were combined with subject headings to improve the retrieval of both indexed and recently published articles. To validate the comprehensiveness of the strategy, keywords and subject headings were refined through a sensitivity check against a gold standard validation set of 5 preidentified articles that met all inclusion criteria.

The search used Boolean logic to combine 3 core concept blocks: (1) AI and ML (eg, “Deep Learning” and “Predictive Model*”), (2) psychiatry and mental health, and (3) continuity of care and adherence (eg, “Care Transition*” and “Readmission*”). Search filters were applied to limit results to English-language primary articles and reviews published from January 1, 2016, to the present.

**Draft search strategy example for PubMed.**

(“Artificial Intelligence”[Title/Abstract] OR AI[Title/Abstract] OR “Deep Learning”[Title/Abstract] OR “ Machine Learning Algorithm*”[Title/Abstract] OR “Predictive Model*”[Title/Abstract]) AND (“Psychiatry”[Mesh] OR “Mental Health” [Mesh] OR “Schizophrenia”[Mesh] OR “Bipolar Disorder”[Mesh] OR “Depressive Disorder, Major”[Mesh] OR “Depressive Disorder”[Mesh] OR schizophrenia*[tw] OR “bipolar disorder*”[tw] OR “major depression*”[tw] OR “psychiatric illness*”[tw]) AND (“Continuity of Care”[Title/Abstract] OR “Continuity of Patient Care”[Title/Abstract] OR “Care Transition*”[Title/Abstract] OR “Assertive Community Treatment”[Title/Abstract] OR “Collaborative Care”[Title/Abstract] OR “Case Management”[Mesh] OR “Case Management”[Title/Abstract] OR “Discharge Planning”[Title/Abstract] “Patient Readmission”[Title/Abstract] OR Readmission*[Title/Abstract] OR Adherence[Title/Abstract] OR Follow-up[Title/Abstract] OR “relapse prevention”[Title/Abstract])

### Study Records and Selection Process

#### Data Management

Following the search, all identified records were exported to Covidence (Veritas Health Innovation) systematic review software for centralized management. To ensure a clean dataset, duplicate records were removed using the software’s automated deduplication algorithm, followed by a manual verification process to identify any remaining duplicate records.

#### Selection Process

The selection of studies followed a rigorous 2-stage process conducted independently by at least 2 reviewers (EJT, WJDY, XEO, JF, IHAP, and MA). In the first stage, reviewers screened the titles and abstracts of all retrieved records in parallel. To ensure interrater reliability, a pilot screening of 20 articles per reviewer pair was conducted with a target agreement threshold of 80%; disagreements during this pilot were analyzed to refine the application of the eligibility criteria.

In the second stage, the full-text documents of all potentially eligible records were retrieved and independently assessed against the inclusion criteria. Throughout both stages, any discrepancies between reviewers were resolved through consensus or, where necessary, through arbitration by a third reviewer. The final results of the selection process, including specific reasons for the exclusion of full-text articles, will be documented and presented in a PRISMA (Preferred Reporting Items for Systematic Reviews and Meta-Analyses) flow diagram.

#### Data Collection

Data will be extracted from the included studies using a standardized data extraction form, which will be developed and prepiloted in a spreadsheet program. To ensure the reliability of the findings and minimize the risk of transcription errors, the extraction will be performed by 2 reviewers independently and in duplicate. Following the extraction phase, the reviewers will meet to compare their findings, and any discrepancies will be resolved through consensus or, if necessary, through arbitration by a third member of the research team. If crucial data—such as SDs or specific outcome values—are missing or insufficiently reported, the authors of the original studies will be contacted via email. A total of 3 contact attempts will be made at weekly intervals over a 3-week period before the study is assessed based solely on the published information or excluded from the meta-analysis.

#### Data Items

The variables to be extracted will be categorized into study characteristics, participant details, and technical and clinical outcomes. Regarding study characteristics, we will record the author, year of publication, country, study design, clinical setting, and funding source. The clinical setting will document the health care tier (eg, primary, secondary, or tertiary) and the precise care settings and direction of transition (eg, inpatient psychiatric ward to primary care and general practitioners, or emergency triage to community teams). To address the PICOS framework, we will extract data on the patient diagnosis, population size, the specific type of AI or ML architecture used (including the algorithm and its primary function), safety fallback pathways with protocols for emergency human-in-the-loop override, a description of the comparator, and the specific outcomes measured. For studies reporting quantitative results, we will extract the means and SDs, incidence rates, relative risks (RRs), hazard ratios (HRs), model sensitivity, specific false-negative rates, undertriage error frequencies, and 95% CIs for all primary and secondary outcomes. Furthermore, for studies involving qualitative components, we will extract relevant themes, categories, and illustrative quotes concerning patient experience and the implementation of the AI technology.

### Risk of Bias in Individual Studies

To ensure the internal validity of the included studies, risk of bias will be assessed independently and in duplicate by 2 reviewers, with disagreements resolved through consensus-driven discussion or arbitration by a third reviewer.

Given that this review centers on the adoption of AI and ML architectures within psychiatric care, the primary framework for critical appraisal will be the Prediction Model Risk of Bias Assessment Tool for Artificial Intelligence (PROBAST+AI) [73]. This tool will be systematically applied to evaluate the development and validation quality, risk of bias, and applicability concerns of the underlying AI algorithms across 4 core domains: participants and data sources, predictors, outcomes, and analysis. If an included study evaluates the clinical efficacy of an AI intervention using an interventional trial design rather than an algorithmic validation framework, the Cochrane risk-of-bias tool (revised Cochrane risk-of-bias tool for randomized trials [RoB 2]) will be used as a secondary tool to assess the randomization process, deviations from intended interventions, missing outcome data, measurement of the outcome, and selection of the reported result.

For studies exploring qualitative adoption barriers, implementation facilitators, or user-experienced phenotypes, the Critical Appraisal Skills Programme (CASP) checklist will be used to ensure the rigor of the research design and reflexivity. Final risk-of-bias judgments will adhere strictly to the grading frameworks native to each tool, incorporating low, moderate, high, or unclear risk of bias alongside corresponding applicability concerns for PROBAST+AI, and will be visually summarized to directly inform data synthesis weights and the overall certainty of the evidence.

### Data Synthesis and Confidence

#### Quantitative and Qualitative Synthesis

To synthesize quantitative efficacy data alongside qualitative experiential findings, this review will use the Joanna Briggs Institute (JBI) convergent segregated approach to synthesis. Quantitative and qualitative data streams will be analyzed independently and then integrated during the narrative discussion to map intervention efficacy directly against human implementation factors. For quantitative data streams, a formal random-effects meta-analysis will be conducted only if a minimum of 3 independent primary studies exhibit clinical homogeneity—specifically evaluating identical stratified AI architectures (eg, predictive risk algorithms) and using uniform clinical indicators. If clinical or methodological heterogeneity is excessive, studies will be synthesized purely using qualitative narrative synthesis following the synthesis without meta-analysis (SwiM) reporting guidelines.

#### Meta-Bias (Assessment of Missing Results)

To ensure the integrity of the cumulative findings, the risk of bias due to missing results will be addressed at both the study and outcome levels. Publication bias will be formally assessed if a minimum of 10 studies are included in a meta-analysis for a specific outcome. Primary studies with irrecoverable variance data will be omitted from quantitative meta-analyses and synthesized exclusively within the narrative framework. For the assessment of publication bias via funnel plot asymmetry, the Egger test and Peters test will be used for continuous and binary outcomes, respectively. If significant asymmetry is detected, the “trim and fill” method will be used as a sensitivity analysis to estimate the potential impact of missing literature on the summary effect size.

Outcome reporting bias will be evaluated at the within-study level by comparing published results against original trial registrations (eg, ClinicalTrials.gov or PROSPERO). Any discrepancies, such as the omission of nonsignificant outcomes or the addition of nonregistered outcomes, will be documented and incorporated into the study’s overall risk-of-bias assessment. Furthermore, if numerical data essential for meta-analysis remain missing after author contact, conservative statistical imputation methods—such as substituting missing SDs with the maximum value observed in other studies—will be used in sensitivity analyses to test the robustness of the findings.

#### Confidence in Cumulative Evidence

The overall certainty of the body of evidence for the primary outcomes will be evaluated using the GRADE (Grading of Recommendations Assessment, Development and Evaluation) methodology. Evidence will be categorized as “high,” “moderate,” “low,” or “very low” based on assessments of risk of bias, inconsistency, indirectness, imprecision, and publication bias. This systematic grading will provide a transparent framework for interpreting the strength of the review’s conclusions.

## Results

**Figure 1:**
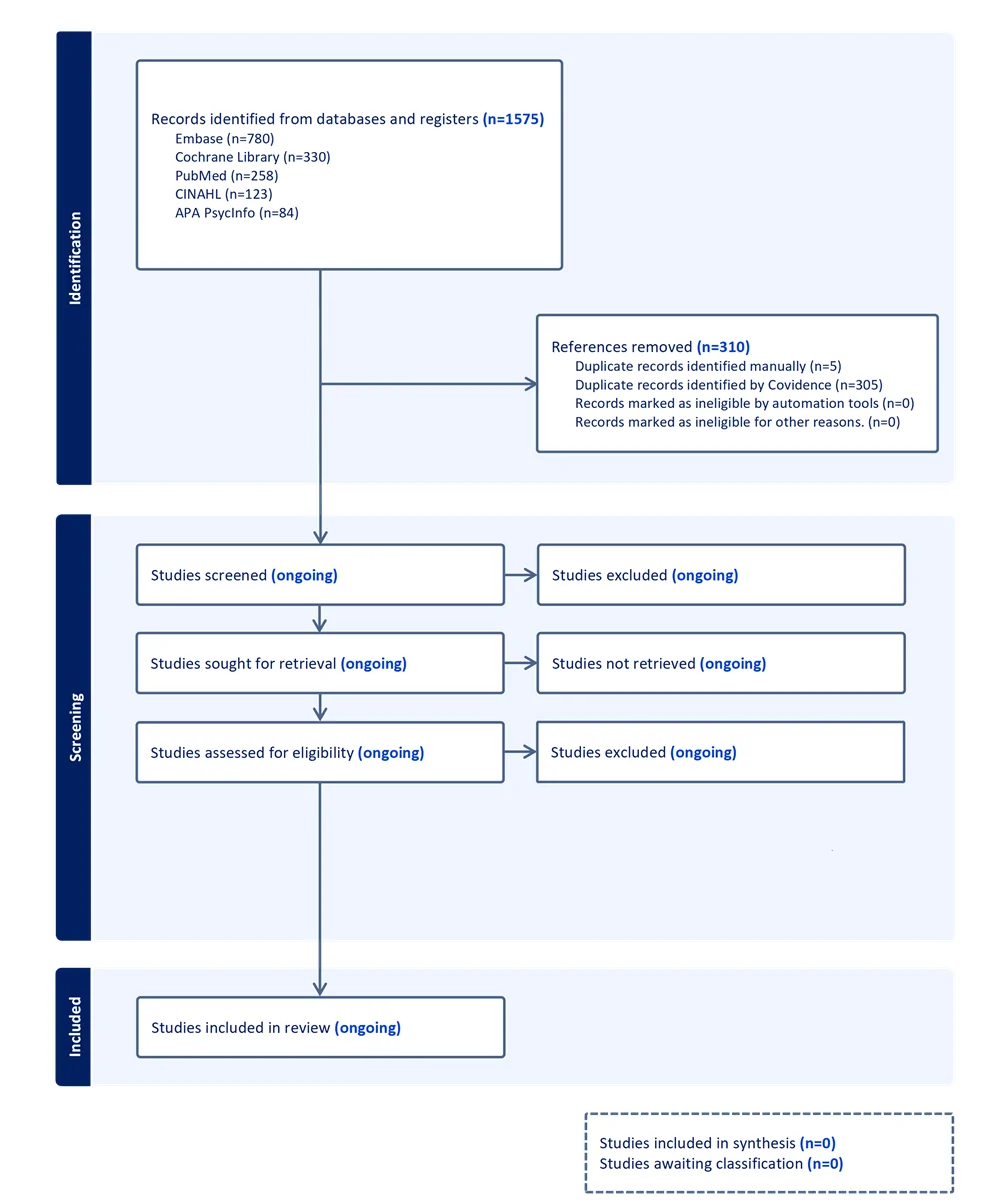
PRISMA (Preferred Reporting Items for Systematic Reviews and Meta-Analyses) flow diagram of the study selection process.

As of June 2026, this systematic review is self-funded and has received no external financial support. The protocol was officially registered with PROSPERO on January 24, 2026. Title and abstract screening are underway, with the comprehensive full-text screening and review of transcripts projected to conclude by August 31, 2026 (Figure 1). Following screening, data extraction, risk-of-bias charting, and specialized quality assessments are scheduled for completion by October 2026. Data synthesis and mixed methods configuration mapping will occur during November 2026 and December 2026, with the compilation and submission of the final systematic review manuscript targeted for early spring 2027. A preliminary PRISMA flow diagram template has been prepared to visually document the article review process.

## Discussion

### Redesigning Psychiatric Care: A Multi-Level Perspective

Continuity of care is a foundational requirement for effective, patient-centered health services. Framing existing and emerging interventions through patient, community, institutional, and system-level lenses clarifies how mental health services can be redesigned to provide sustained, coordinated support rather than episodic, crisis-driven care [74].

### Patient Level: Digitally Extended Therapeutic Contact

Remote monitoring models are increasingly being adapted to mental health services, particularly for individuals with comorbid medical conditions [75-78]. These programs combine in-home physiological monitoring, symptom check-ins, and telepsychiatry contacts to maintain close observation following discharge, with early evidence suggesting reductions in readmissions and improved treatment adherence when such support is provided [79]. For patients, this translates into a more continuous therapeutic relationship and a clearer sense that care extends into their everyday environments rather than being confined to traditional facility-based medicine.

“Digital bridge” approaches using enhanced patient portals and mobile apps are also highly relevant for psychiatric care. In somatic specialties, portals already allow patients to upload wound photos or blood pressure logs that feed into clinician dashboards; analogous tools in mental health can host mood diaries, sleep and activity logs, PROs, and safety plans [80-82], enabling asynchronous 2-way communication between patients and their care teams [83,84]. When integrated into existing workflows, such tools support informational continuity by ensuring that clinicians have up-to-date, patient-generated data on symptoms, functioning, and side effects, while patients gain a stable, accessible channel for help seeking between scheduled visits. Emerging models also incorporate structured relapse prevention plans, crisis contacts, and psychoeducation modules within portal interfaces, reinforcing self-management and a shared understanding of care plans across settings [85-88].

### Institution and Community Level: Outreach and Home-Based Mental Health Support

Services such as “hospital-at-home” and community paramedicine have illustrated how in-home outreach can sustain continuity after discharge [89-93]. Although initially developed for medical conditions, these programs show that scheduled home visits for recently discharged patients—encompassing assessment, medication reconciliation, and basic support—can reduce emergency department use and improve linkages to care [94-96]. Adapting this approach to mental health involves using community psychiatric nurses, case managers, and peer support workers, sometimes augmented by telehealth, to provide early postdischarge contact, monitor early warning signs of relapse, and address social determinants of health such as housing instability or social isolation [96,97].

Other community-level mental health programs similarly aim to bridge fragmented services. Assertive community treatment through intensive case management [98-101] or the Naylor transitional care model provide multidisciplinary outreach to individuals with severe and persistent mental illness, emphasizing shared caseloads, 24-7 crisis response, and home-based care to avoid hospitalizations and maintain engagement [102-106]. Collaborative care models in primary care integrate care managers and consulting psychiatrists to support patients with depression and anxiety over time, using registries and systematic follow-up to ensure that no patient “falls through the cracks.” Together, these approaches enhance relational continuity by providing stable contacts in the community and management continuity by aligning roles across mental health, primary care, and social services.

### Health System Level: Payment and Policy Levers for Continuity

Redefining quality and safety within financing and regulatory frameworks will support continuity of mental health care at the health system level [107-111]. The shift from fee-for-service to value-based care and population-based payment models encourages providers to invest in team-based, longitudinal approaches rather than short, high-volume encounters. These include financial incentives that enable practices to fund care coordination, outreach, and the integration of behavioral health without relying on visit-based billing. In the psychiatric context, such models incentivize management continuity by making it financially advantageous to keep patients stable in the community, maintain regular follow-up, and prevent crisis admissions.

Against this backdrop, the integration of AI across these levels offers substantial potential to address persistent gaps in psychiatric continuity of care. Yet, these opportunities are not without challenges, which this systematic review is well positioned to clarify. The proposed review will therefore make an important contribution by systematically synthesizing how AI has been used to enhance continuity of care within mental health systems, mapping interventions across patient, community, institutional, and system levels. By characterizing target populations, continuity constructs, outcome measures, and implementation contexts, the review will help to delineate which AI-enabled strategies show promise; where evidence remains sparse; and how future work should be designed to address equity, safety, and acceptability.

### Strengths and Limitations

A primary structural limitation of this protocol is the explicit exclusion of pediatric and adolescent populations. Although this boundary is methodologically necessary to maintain a clinically homogeneous assessment of adult-centered AI deployment models and eliminate complex caregiver confounding variables, it represents a notable limitation. The transition interface from pediatric to adult psychiatric services represents a high-risk period for care fragmentation, and future reviews must explicitly evaluate AI’s efficacy within this specific transitional population. Ultimately, clarifying the role of AI within this multilevel continuity framework may inform the development of integrated, learning mental health systems that are proactive; person-centered; and capable of providing sustained, coordinated care across the life course.

## Appendix

Multimedia Appendix 1 PRISMA-P checklist.

## Abbreviations

CASP: Critical Appraisal Skills Programme
GRADE: Grading of Recommendations Assessment, Development and Evaluation
HR: hazard ratio
ICTRP: International Clinical Trials Registry Platform
JBI: Joanna Briggs Institute
ML: machine learning
PICOS: population, intervention, comparator, outcome, and study design
PRISMA: Preferred Reporting Items for Systematic Reviews and Meta-Analyses
PRISMA-P: Preferred Reporting Items for Systematic Review and Meta-Analysis Protocols
PRO: patient-reported outcome
PROBAST+AI: Prediction Model Risk of Bias Assessment Tool for Artificial Intelligence
RoB 2: revised Cochrane risk-of-bias tool for randomized trials
RR: relative risk
SwiM: synthesis without meta-analysis
